# Bridging the gap between pregnancy loss research and policy and practice: insights from a qualitative survey with knowledge users

**DOI:** 10.1186/s12961-024-01103-z

**Published:** 2024-01-25

**Authors:** Marita Hennessy, Keelin O’Donoghue

**Affiliations:** 1 Pregnancy Loss Research Group, Department of Obstetrics and Gynaecology, University College Cork, Cork, T12 YE02 Ireland; 2 INFANT Research Centre, University College Cork, Cork, T12 YE02 Ireland

**Keywords:** Communication, Evidence-based practice, Health policy, Knowledge translation, Miscarriage, Pregnancy loss, Qualitative research, Qualitative survey, Stigma, Stillbirth

## Abstract

**Background:**

The loss of a pregnancy or the death of baby around the time of their birth can have profound impacts on parents, families and staff involved. There is much opportunity to enhance the systematic uptake of evidence-based interventions to enhance service provision, lived experiences and outcomes. Challenges exist to translating pregnancy loss research evidence into policy and practice, however. Pregnancy loss remains a neglected area of research and resourcing and is steeped in stigma. While barriers and facilitators to the use of research evidence by decision-makers in public health and health services are well documented, we aimed to better understand the factors that influence the translation of pregnancy loss research into practice and policy.

**Methods:**

We conducted a qualitative online survey of pregnancy loss research knowledge users in Ireland, identified through our clinical and academic networks, between January and March 2022. The survey comprised ten questions, with three closed questions, informed by the Knowledge Translation Planning Template©. Questions included who could benefit from pregnancy loss research, perceived barriers and facilitators to the use of research evidence and preferred knowledge translation strategies. We analysed data using reflexive thematic analysis.

**Results:**

We included data from 46 participants in our analysis, from which we generated two central themes. The first—‘End the silence; stigma and inequality around pregnancy loss to enhance awareness and understanding, public health and services and supports’—addresses issues related to the stigma, sensitivities and silence, lack of awareness and understanding, and lack of relevance or priority afforded to pregnancy loss. The second theme—‘Use a range of tailored, accessible approaches to engage a large, diverse range of knowledge users’—highlights the need to use relevant, accessible, and engaging information, resources or materials in knowledge translation efforts, and a variety of tailored approaches to suit different audiences, including materials, workshops/webinars, media, knowledge brokers and champions or opinion leaders.

**Conclusions:**

Our analysis provides rich insights into the barriers and facilitators to knowledge translation in the field of pregnancy loss research. We identified key strategies that can be used to inform knowledge translation planning in Ireland, and which have international applicability.

**Supplementary Information:**

The online version contains supplementary material available at 10.1186/s12961-024-01103-z.

## Background

Pregnancy loss, in the form of miscarriage, stillbirth or neonatal death, occurs in 20–25% of all pregnancies. It is estimated that at least 15% of clinically recognized pregnancies miscarry [1], including up to 3% of all pregnancies which end in second trimester miscarriage [2], while the risk of stillbirth is 3.5 per 1000 total births [3]. Pregnancies can also end in termination or abortion; impacting approximately 39 per 1000 women aged 15–49 years [4]. The loss of a pregnancy, or death of a baby around the time of birth, can have profound physical, psychological and economic impacts on individuals and society [1, 5]. Despite many improvements in medical care, progress has been slow in reducing stillbirth rates [3, 6, 7]. Indeed recent research shows that stillbirth rates are increasing in some high-income countries, including Ireland [8], England and Wales [9] and Germany [10], and rates of miscarriage are also increasing in some countries [1]. The need for action to address issues relating to miscarriage and stillbirth has been highlighted internationally [11, 12].

There is much opportunity to enhance the systematic uptake of evidence-based interventions in the field of pregnancy loss, and within maternity care more broadly [13]. For example, clinical practice guidelines are often not implemented fully in practice [14–17], despite the potential to reduce stillbirths and perinatal mortality [18, 19]. Internationally, care experience surveys demonstrate that there are aspects of care which are sub-optimal and not aligned with evidence-informed care [20–22]. Public knowledge concerning the incidence, risk factors and causes of various forms of pregnancy loss is limited [23–26], with calls for the implementation of evidence-based interventions to enhance public awareness and knowledge [8, 27, 28]. The UK Stillbirth and Miscarriage Priority Setting Partnerships have also identified issues surrounding communication and awareness as research priorities [29, 30]. Progress in enacting legislation and implementing change concerning pregnancy loss has been slow, occurring against a backdrop of cultural and religious influences on reproductive rights [31]. Stigma around pregnancy loss—including stillbirth [32–34], miscarriage and abortion [35, 36]—is one of the main barriers in improving outcomes and care experiences and has been the subject of many calls for action [37].

It is frequently cited that it can take 17 years for research evidence to reach practice [38], and although contested, this gap persists [39]. Knowledge translation is a ‘dynamic and iterative process that includes synthesis, dissemination, exchange and ethically sound application of knowledge to improve health’ [40]. Dissemination aims to increase awareness, knowledge, perceptions or motivation (antecedents of behaviour change) by strategically communicating information to target knowledge users—individuals who are likely to use and benefit from research results to inform their decision making around practices, programmes or policies [41]. Ideally, dissemination precedes implementation, which aims to promote the enactment of specific behaviours [42]. It is most effective when it starts early, stimulates support, uses champions and brokers, considers contextual factors, is timely, relevant and accessible, and knows the players and processes [43]. Knowledge translation strategies—encompassing dissemination and/or implementation—can facilitate the uptake of evidence into policy and practice, targeting change at individual, institutional or policy levels. There has been a growing number of knowledge translation interventions, as well as frameworks, theories and models to guide the selection of knowledge translation strategies in recent decades [44–47]. However, the range of knowledge translation strategies related to pregnancy loss practice and policy improvements remains unknown.

Research to understand influences on the use of pregnancy loss research in policy and practice is limited and is needed to inform knowledge translation strategies. Influences on the use of research evidence by decision makers in public health and health services are well documented. Barriers include the lack of relevant research, perceptions of evidence and skills and opportunity to use it, the culture and competing demands surrounding decision making, as well as practical constraints such as time and cost [48, 49]. Enablers include access to and improved dissemination of relevant research, as well as promoting collaboration between policymakers and research staff [48]. Such influences have not been studied, specifically in relation to pregnancy loss, with decision makers or indeed other types of knowledge users. To increase knowledge translation efforts, we first need to understand the barriers and facilitators to uptake/adoption of pregnancy loss research in practice and policy.

Through an involvement activity with knowledge users, we aimed to better understand the factors that influence the translation of pregnancy loss research into practice and policy. Our objectives were to: (1) understand barriers and facilitators to research use and (2) identify preferred knowledge translation strategies. We focus on a broad range of knowledge users who can use pregnancy research loss to inform decision making at micro (individual or clinical), meso (organizational) and macro (regional or national) levels. In our analysis we seek to explore any variation in perceived influences on knowledge translation and preferred knowledge translation strategies.

Throughout this article, we use terms such as ‘pregnancy loss’, ‘miscarriage’ and ‘parents/parent advocates/bereaved parents’; however, we recognize that people have different views on the appropriateness of such terminology [50–52], particularly when applying it across different types of pregnancy loss experiences.

## Methods

We utilized a qualitative online survey (available in Additional file 1) to better understand the factors that influence how pregnancy loss research can, or indeed should, influence policy and practice from the perspectives of a variety of pregnancy loss research knowledge users, including health professionals (hospital- and community-based), parent advocates/bereaved parents, support group representatives, journalists/media representatives, academics/researchers, medical students and decision makers. We draw on the Standards for Reporting Qualitative Research [53] in reporting our involvement activity (see Additional file 2).

### Theoretical framing

Our work is concerned with eliciting knowledge users’ perceptions of what works, or not, and is informed by social constructionism and theories and frameworks in knowledge translation [44–46]. We approached this activity through the lens of social constructionism, acknowledging that there can be multiple perspectives on issues, events and activities—in our case, knowledge translation and pregnancy loss where such knowledge is socially constructed, culturally and historically situated, and dominant perspectives can arise from political and power relations [54]. Supported by the Knowledge to Action Framework, we interpreted knowledge translation from the creation of knowledge to its application [44]. We also attended to constructs from the Integrated-Promoting Action on Research Implementation in Health Services (i-PARIHS) framework which proposes that successful implementation of an innovation is the result of the facilitation of an innovation with recipients (individual and collective) in their context (inner and outer) [45, 46].

### Qualitative survey

We developed a qualitative survey, drawing on elements of the Knowledge Translation Planning Template© [55], to achieve our aim. Qualitative surveys give participants control regarding their research participation and are generally less burdensome than in-person interviews as participants can complete them at a time, and in a location, that suits them; they also negate the need for transcription [56]. The Knowledge Translation Planning Template^©^ enables the planning of a knowledge translation strategy [55]. It incorporates various steps including the identification of knowledge users, main messages and knowledge translation goals and strategies. The lead author was trained in the use of this framework as part of the SickKids Knowledge Translation Professional Certificate™.

The survey included questions relating to: who could benefit from pregnancy loss research and reasons for this, perceived barriers and facilitators to the use of research evidence, whether pregnancy loss research is any easier or harder to get into policy/practice than other health topics, or indeed within the field itself, and preferred knowledge translation strategies for pregnancy loss research (see survey questions in Additional file 1). We also asked participants what information they would like to see in a website that we were developing for our group (The Pregnancy Loss Research Group) [57]. We invited any additional comments at the end. We administered the survey via Qualtrics software [58].

We did not force responses to any of the questions so that participants could move freely through the survey and respond (or not) to any questions. We included two closed questions to prompt participants’ thinking around knowledge user and knowledge translation strategies based on the Knowledge Translation Planning Template© (a further closed question focused on participant characteristics), and we provided prompts for each question, including encouraging participants to tell us as much as they could in their responses; these were attempts to mitigate the aforementioned limitations of qualitative surveys. We encouraged participants to think about their own views or needs and to reflect on how their colleagues, peers or others might respond to the survey questions also, as they formulated their responses. We piloted the survey with members of the Pregnancy Loss Research Group prior to administration; no changes were made.

### Participants and recruitment

We invited, by email, a pre-determined selection of knowledge users that we frequently engage with individually and/or through various fora (*n* = 87) and members of the Pregnancy Loss Research Group (*n* = 30) to participate in the survey between January and March 2022, with reminders. The former included members of the RE:CURRENT Research Advisory Group [59] and Oversight Group for the National Standards for Bereavement Care Following Pregnancy Loss and Perinatal Death [60], and other health professionals, media representatives, decision-/policy-makers and parent advocates. The Irish Hospice Foundation [61], our project partners, also shared details of the survey with its staff members (*n* = 40) and members of the Irish Childhood Bereavement Network [62] Steering Group (*n* = 12). All knowledge users were provided with the same, standardized information about the activity. We kept the survey open until a range of knowledge users had participated and we felt that we had dataset richness and sufficiency to address our aims [56].

### Data analysis

Data were downloaded from Qualtrics into Microsoft Excel and imported into NVivo for data management and analysis. We analysed the quantitative data descriptively in Excel, reporting frequencies for responses to closed questions. We analysed responses to open-ended questions following the six phases of reflexive thematic analysis advocated by Braun and Clarke to identify patterns of meaning within the data; this analytical approach is theoretically flexible [63]. We familiarized ourselves with the survey responses by reading and re-reading them. We then coded responses to each individual question, developing initial themes, before generating overall themes across the dataset. We reviewed these themes in terms of their relationship to our aims, each other and to the dataset. We did not code according to a predefined framework, but rather engaged in primarily inductive coding at semantic (surface meaning) and latent (underlying meaning) levels. Because the analysis was underpinned by social constructionism, we did not take the participants’ accounts as face value, instead adopting a critical lens to look beyond the data surface to interrogate and interpret their accounts. M.H. led the analysis, with ongoing discussions of codes and themes with K.O.D.

### Ethical considerations

This was an involvement activity—facilitated via a qualitative survey—with key knowledge users that we frequently engage with to inform knowledge translation activities/efforts rather than a research activity per se; therefore, ethical approval was not required [64]. We provided all participants with information about the activity and what it would involve before they agreed to participate (see survey information sheet; Additional file 1).

### Reflexive statement

M.H. is postdoctoral researcher, with expertise in public health, health services research, dissemination and implementation science, and qualitative research. She has been researching in the field of pregnancy loss since 2020. K.O.D. is a consultant obstetrician and maternal fetal medicine sub-specialist. She founded the Pregnancy Loss Research Group in 2012 and has been active clinically and academically in the field of pregnancy loss for almost 20 years. Both are committed to and actively engaged in efforts to close the research to practice gap and engaging knowledge users—including people with lived experience—in such efforts. We work and/or collaborate with the knowledge users invited to participate in this involvement activity, or have in the past.

## Results

We received 57 responses between 24 January and 15 March 2022, of which 46 (81%) were deemed eligible for inclusion in our analysis based on participants providing a response to at least one question (‘Which of the following best describes you’ excluded).

### Participant characteristics

The majority of participants identified as ‘Health professional—hospital-based’ (*n* = 11, 24%), ‘Academics—medical/nursing/midwifery’ (*n* = 5, 11%) and ‘Researchers (including PhD students)’ (*n* = 5, 11%) (Table 1).

**Table 1:** Participant roles: combined roles

Role	*n*	% (*N* = 46)
Health professional—hospital-based	11	24
Academic—medical/nursing/midwifery	5	11
Researcher (including PhD students)	5	11
Health professional—community/primary care	4	9
Parent advocate/bereaved parent	3	7
Parent advocate/bereaved parent; support group representative	3	7
Other [Advocate for quality end-of-life care; Librarian; Marketing professional]	3	7
Academic—medical/nursing/midwifery; health professional—hospital-based	2	4
Health professional—hospital-based; researcher (including PhD students)	2	4
Journalist / media representative	2	4
Academic—social sciences; health professional—community/primary care; parent advocate/bereaved parent; support group representative; researcher (including PhD students)	1	2
Academic—social sciences; health professional—community/primary care; researcher (including PhD students)	1	2
Decision maker (a person with power to influence or determine policies and practices at local, regional, national or international level)	1	2
Decision maker (a person with power to influence or determine policies and practices at local, regional, national or international level); health professional—hospital-based	1	2
Journalist/media representative; parent advocate/bereaved parent	1	2
Medical student	1	2
*Total*	*46*	*100*

As highlighted above, some participants had more than one role or identity. When individual roles were considered, the majority of participants identified as ‘Health professional—hospital-based’ (*n* = 16, 35%), ‘Researcher (including PhD students)’ (*n* = 9, 20%), ‘Parent advocate/bereaved parent (*n* = 8, 17%)’, Academic—medical/nursing/midwifery (*n* = 7, 15%) and Health professional—community/primary care (*n* = 6, 13%) (see Table S1, Additional file 3).

### Quantitative analysis: priority knowledge users and preferred knowledge translation strategies

Two closed questions were asked to provide similar response options across the data set and prompt participants’ thinking. Four particular categories of knowledge user that could benefit from knowing about pregnancy loss research were prioritized by 80% or more of all participants (*N* = 46): women/men with lived/living experience of pregnancy loss (*n* = 41, 89%), policy makers/government (*n* = 40, 87%), decision makers (*n* = 39, 85%), media (*n* = 39, 85%), members of the public (*n* = 39, 85%) and practitioners/service providers (*n* = 37, 80%) (Table 2).

**Table 2:** Who could benefit from knowing about pregnancy loss research

Knowledge user	*n*	% (*N* = 46)
Women/men with lived/living experience of pregnancy loss	41	89
Policy makers/government	40	87
Decision makers (people with power to influence or determine policies and practices at local, regional, national or international level)	39	85
Media	39	85
Members of the public	39	85
Practitioners/service providers^a^	37	80
Research funders	29	63
Researchers	27	59
Volunteer health sector/NGOs	27	59
Private sector/industry	13	28
Other—please specify [teachers/educators]	2	4

^a^Thirteen participants specified further; these included any or all health and allied health professionals who engage with pregnant women and/or general women’s health—obstetricians/gynaecologists, nurses, midwives, staff in emergency departments, primary care practitioners (general practitioners, practice nurses and public health nurses) and counsellors and psychotherapists

A total of 33 participants (72%) responded to the question regarding how they would like pregnancy loss research to be shared with them. A wide range of knowledge translation strategies (from the pre-defined list of strategies from the Knowledge Translation Planning Template©) was endorsed; the most popular being: materials (*n* = 24, 73%), workshops, including webinars (*n* = 24, 73%), media (*n* = 22, 67%), knowledge brokers (*n* = 21, 64%), peer-reviewed publications, i.e. journal articles (*n* = 21, 64%), champions/opinion leaders (*n* = 20, 61%) and conferences (*n* = 20, 61%) (Table 3).

**Table 3:** Preferred knowledge translation strategies

Knowledge user	*n*	% (*N* = 33)
Materials (guides/toolkits/pamphlets)	24	73
Workshops, including webinars	24	73
Media	22	67
Knowledge brokers (individuals who link decision makers and researchers, and facilitate the use of research-based evidence in decision making)	21	64
Peer-reviewed publications, i.e. journal articles	21	64
Champions/opinion leaders	20	61
Conferences	20	61
Leadership (through leaders who can foster/facilitate change and innovation)	19	58
Consultants (medical)	18	55
Professional development	17	52
In-service training	17	52
Social media Please specify any particular channels (*n* = 10): Twitter (7), Instagram (7), Facebook (2), LinkedIn (1), all (1)	17	52
Networks, communities	16	48
Policy briefs	16	48
Collaborations/partnerships	14	42
Grey literature, e.g. reports, working papers	12	36
Stakeholder position papers	11	33
Arts-based strategies, e.g. visual arts, performing arts, creative writing, multimedia-including video and photography	10	30
Meeting dialogue	8	24
Consultants (non-medical, e.g. business, innovation, marketing)	5	15
Science policy fellowships, placements	5	15
Other Please specify: through voluntary organisations that deal with pregnancy loss	1	3

### Qualitative analysis

We actively generated two themes from participants’ responses across the entire dataset: (1) end the silence; stigma and inequality around pregnancy loss to enhance awareness and understanding, public health and services and supports and (2) use a range of tailored, accessible approaches to engage a large, diverse range of knowledge users. These themes, and sub-themes, are presented in Table 4. Participant identities accompany the illustrative quotes; however, to preserve anonymity, we have not included participants’ roles.

**Table 4:** Themes and sub-themes

Theme	Sub-themes
End the silence, stigma and inequality around pregnancy loss to enhance awareness and understanding, public health and services and supports	• Steeped in stigma, sensitivities and silence • Lack of awareness and understanding • Not seen as relevant, or a priority
Use a range of tailored, accessible approaches to engage a large, diverse range of knowledge users	• Accessible, engaging, relevant information • Variety of tailored approaches needed

#### Theme 1 | End the silence, stigma and inequality around pregnancy loss to enhance awareness and understanding, public health and services and supports

Within this theme, three interlinked sub-themes around pregnancy loss and inherent challenges to reducing the knowledge to policy and practice gap are illuminated: stigma, sensitivities and silence, lack of awareness and knowledge, and perceived lack of relevance or priority afforded to the area.

##### Steeped in stigma, sensitivities and silence

Many highlighted how stigma surrounding pregnancy loss impacted on knowledge translation efforts: it is a ‘sensitive/taboo topic that can be difficult to discuss/incorporate into policy’ [P5]. The sensitive nature of pregnancy loss and societal struggles around grief and bereavement result in a lack of public and private discourse around these issues, by people with and without lived or living experience.

‘The public are steeped in ‘its private/shameful/woman’s business/embarrassing’. People with experience are vulnerable and don’t feel they have acceptance or permission to talk about it (should keep it quite)…… For many they may be avoidant to avoid their own experience of loss’ [P15]

‘In the broader public and in education (school/university)—an historic and deep routed culture of stigma and tabus whereby issues around female health, pregnancy, fertility etc. are not discussed or when they are the impact of bias (related to religion, cultural views, gender etc.) is deep’. [P14]

Some also highlighted reluctance amongst researchers to ask people about loss, being ‘too afraid of upsetting people’ [P15], and the resulting ‘unintended consequence here of silencing the bereaved’ [P28]. Many stated that they felt that pregnancy loss research was more difficult to get into policy and practice than other topics:

‘It’s an emotive topic. It makes people uncomfortable. It’s still a hidden grief and quiet taboo. As such I think it is harder to put the research into practice. It’s not always seen as a priority... It’s not always recognized the huge impact pregnancy loss has. The research may not be seen as important and may be pushed to the back of the line’ [P19].

A few noted that certain types of pregnancy loss may be easier to get into policy/practice, including later losses (e.g. stillbirth) and/or fatal fetal anomalies which, although ‘less common’ than miscarriages, can be perceived as ‘real losses’ or involve tragic or shocking aspects which will ‘grab media headlines but usually after women/couples have been mistreated or let down by systems’ [P5]. Earlier losses, such as miscarriages and molar and ectopic pregnancies, might not be perceived as important or ‘valid’, although ‘common’ or ‘not common enough or as real baby losses’ [P15]. One participant also noted how this can impact what research is conducted and/or whose voices are heard, contending that there is ‘not enough research on lived experience of parents, especially where baby has a life-limiting condition’ [P33]. This ties in with the stigma surrounding, and indeed within, pregnancy loss.

##### Lack of awareness and understanding

Participants perceived that there was a general *lack of awareness and understanding of pregnancy loss* amongst many knowledge users, who are ‘removed from clinical care and lived experience’ [P8]. This extends to the various types of pregnancy loss, and the impact on women and men who experience the loss of a pregnancy, as well as society more broadly, including ‘the economic impact of loss of productivity to society associated with loss’ [P2]. As such, participants felt that a broad range of knowledge users needed to be targeted and would benefit from knowing more about pregnancy loss research. If more people knew about the nature of pregnancy loss and its impacts (and were interested/invested), they believed that it would be easier to affect change in this area (including policy, practice, public and private discourses) for a variety of reasons, including that it would be harder to ignore the evidence, impacts and need. Many felt that raising awareness would reduce stigma and isolation, enhance knowledge and preventive efforts, challenge misconceptions around pregnancy loss and improve the provision and quality of care and supports—within families, communities, society, healthcare, policy and beyond.

‘For decision/policy makers, if they were more aware of the personal impact of pregnancy loss, it might lead to the development of more empathetic policies e.g. around leave for miscarriage, opening up the stillbirth registry, funding for bereavement supports etc. For media, knowledge of the latest research/best practice recommendations can lead to news/features to raise awareness of issues experienced by those who suffer loss and fuel an ongoing conversation to make this topic less taboo so that people feel less alone, that they know there is support etc. For groups like Feileacain [the Stillbirth and Neonatal Death Association of Ireland] etc, it’s important to know the research around standards of care etc. and what good practice looks like in order to advocate for those they represent. For those with lived experience, I think it’s important to know research around the physical and psychological impact of pregnancy loss so they understand what they are going through, and also research around best practice so that they can advocate for themselves and their babies for the best care possible. For members of the public, unless personally impacted, they may be less interested in research, but a basic awareness of the main research, how common pregnancy loss is etc. can lead to better understanding and compassion in society at large. This could also apply to private industry for better HR policies and support strategies in the workplace’ [P34].

‘Awareness around pregnancy loss is needed for the general public. The general public don’t see it as an issue until it affects them personally… More research funding should be provided to this area to improve services, information and awareness. Decision maker and policy makers ned to understand the impact that pregnancy loss can have on HCPs [healthcare professionals] and couples’ [P6].

Some also spoke about how women and men with lived experience need to know what research is being done, how they can get involved and its benefits (for themselves directly, and/or others in the future); it can also make them ‘feel heard’, ‘help them cope’ [P9] and ‘validate their loss’ [P19].

##### Not seen as relevant, or a priority

For many, pregnancy loss was ‘a neglected area for many years now’ [P8] in practice and policy, despite its prevalence and wide-ranging impacts. It was perceived as an area that was *not seen as relevant or a priority* by various knowledge users, including members of the public, women during pregnancy who may ‘consider it upsetting or not relevant to them’ [P11], bereaved parents who may be overcome with grief, or policy makers, health professionals and others who may have little awareness of, or interest in, the area. Participants noted how the public may not see the topic as relevant to them, unless they experienced pregnancy loss themselves, and therefore do not engage with it. Other knowledge users may also not recognize the relevance of the topic and/or not have a good level of awareness or knowledge around it.

‘Harder, in part because the financial impacts/losses are not ‘palpable’ (i.e. the economic/financial impact on the services) and in part because of the cultural issues (mentioned before) around this which lead to a mentality whereby pregnancy loss is not seen as a priority or an issue of as much relevance as others. It is also seen mostly as a female problem and this is closely related to cultural issues around how aspects of female health are addressed/seen and/or prioritized. An overall lack of awareness on the magnitude of the problem and its impact and knock-on effect is one of the main reasons why this a challenging topic to include into policy/practice discussions’ [P14].

Within funding and healthcare, ‘big topics like cancer and cardiovascular disease [were seen to] tend to dominate’ [P12] making it more difficult to get pregnancy loss on the agenda, with priorities shifting based on temporal political and social agendas.

‘Reproductive justice, women’s health is topical at present. Trauma and loss may well get more recognition in future. Health is often driven by short term targets of numbers—deliveries, waiting lists. Metrics that reflect compassionate or quality of care only lately being used. The long-term outcome both psychologically and financial (litigation) are less connected in the funding system’ [P26].

Participants highlighted that time and resources were needed to disseminate knowledge and implement appropriate evidence-based care (including care pathways) and supports within healthcare provision and workplaces, but were limited. Pregnancy loss had to compete against other areas and ‘so many vying for funds. Time, nothing happens quickly and things can frazzle out and lose traction’ [P19]. Without the necessary resources, knowledge translation would ‘increase the burden on the services already in place’, [P28] and this participant also noted ‘research burn-out’ amongst people who were ‘constantly taking part in research projects but witnessing change and improvements so slowly’.

Lack of visibility in research funding, and fatal determinism—not seeing or believing that there are solutions to prevent pregnancy loss resulting in inaction (especially amongst the general public, media and policy/decision makers)—were also cited as knowledge translation barriers.

‘Pregnancy loss is an emotive topic and I think that is one which is also accompanied by a sense of inevitability or nihilism. It is a contradictory area of research because although it is well known, often experienced (in private in many cases), it is not widely understood. Members of the public and key Government decision makers may feel there is not much that can be done as they do not understand how these can be prevented, that people need support while experiencing pregnancy loss and also the crucial (for Government) economic impact pregnancy loss may have’ [P18].

Some participants mentioned that sexism/inequality and ‘our paltry approach to women’s health and reproductive health in general’ [P2] have made it difficult to translate pregnancy loss research into policy and practice: ‘It is also seen mostly as a female problem and this is closely related to cultural issues around how aspects of female health are addressed/seen and/or prioritized’ [P14].

#### Theme 2 | Use a range of tailored, accessible approaches to engage a large, diverse range of knowledge users

This second theme turns attention to the ways in which pregnancy loss research can be communicated, and indeed conducted, to enhance its translation into policy and practice. Participants emphasized the importance of accessible, engaging and relevant information, as well as the use of a variety of tailored approaches to meet the needs of a broad range of knowledge users.

##### Accessible, engaging and relevant information

The need for *relevant, accessible and engaging information, resources or materials* featured across all participants’ accounts. This related to ensuring that information is: tailored to the particular knowledge user or audience being targeted, including language and format (sensitive and culturally and linguistically appropriate); relevant and presented clearly and concisely; accessible in terms of people being able to easily find or access it (e.g. shared through multiple channels and/or channels that they use, including standard protocols and guidelines, training, support groups and social media); made relevant or relatable through the sharing of lived experiences or personal stories, and by showcasing positive impacts or outcomes.

‘The key component I think in using research evidence is to be told about it. Most of us want to do the right thing by the people we serve, whether in the business world, professionally in the health services, as family members/friends of the affected, or policy makers. Well researched and clear evidence with clear outcomes informs us as a society—but getting it out there in competition with so much else going on is a challenge’ [P28].

‘Facts and figures that are easy to understand and relate to social, economic and environmental factors (among others). Real life stories and experiences can illustrate impact of whatever is trying to be conveyed. Imagery and graphics that can help people to see and understand complex information or vast amounts of it’ [P18].

‘Dissemination in an accessible manner with plain English summaries, trusted sources disseminating the clinical significance of the research to healthcare practitioners to save them time in assessing the quality of the research. Development of pathways and guidelines for healthcare professionals so a national standard of structured care can be developed to ensure all healthcare practitioners follow the best practice’ [P38].

Participants discussed how research can be inaccessible on several levels—including characteristics of individuals, the research itself and structures in which knowledge is produced and shared. Examples of issues surrounding accessibility included: not knowing where to source and/or how to interpret and apply research, journal articles being behind paywalls, research written in a way that is not easily understood (use of jargon) and/or without being explicit about its significance (on a personal level, or clinically), and the inability to access up-to-date research or research relevant to a person’s needs, to inform decision making. Participants suggested that ensuring that research has been rigorously conducted, so that people can have confidence and/or trust in the findings, is important. A few also mentioned that it is important that knowledge users, particularly those with lived experience, are involved or engaged in the research process.

##### Variety of tailored approaches needed (to suit particular audiences)

Participant accounts demonstrated that tailored knowledge translation strategies are needed to suit particular knowledge user groups, including both the communication channel and the message/information being shared; ‘for each stakeholder group there needs to be a segregated approach to delivering the messages (sometimes the messages are the same but just told differently)’ [P18]. In general, participants felt that certain strategies were suited to certain audiences. For example, the public may engage more with media, materials, workshops, social media and/or online information; researchers, health professionals and policy makers may engage more with policy briefs, opinion leaders, conferences and/or peer-reviewed journals; those in primary care and hospitals settings may prefer pamphlets/leaflets. Many felt that use of a wide variety of strategies was needed to broaden engagement, and increase awareness and knowledge, as one size did not fit all and people needed to be pro-actively engaged in a variety of ways.

‘One area for consideration is the harnessing of culturally appropriate approaches for those who do not engage with the usual health messaging fora’ [P2].

‘Regardless of what your role is, you learn in a way that is unique to you, so I think we need a good mix of materials and ways of teaching in order to capture the interest in the issue of all those involved… once you know it, you can use it’ [P28]

‘I think that media/social media/art-based strategies (e.g. movies, creative writing etc.) have the potential to normalize the topic for the masses… Research is important to come from the medical community e.g. consultants, conferences, workshops etc. to provide continued education and to ensure our providers are informed on a sensitive [topic]’ [P9].

‘I think the human interest/personal approach works best for those with lived experience and media. Media articles, using social platforms like Instagram, collaborations with charities like [Charity Name] who are already engaged with patients/families, I think these are all good ways to reach people who would not be in the academic sphere, reading medical journals etc. You can see in the UK… how they [campaigns] use social media to get their message out. It’s user friendly, accessible, personal, but also sensitively done’ [P34].

A few also noted that it should be easier to get pregnancy loss research into policy/practice through champions—people with an interest and commitment within healthcare, advocacy organizations (including individuals with lived experience not necessarily affiliated to any organization) and policy/decision making—advocating for change. ‘Healthcare practitioners recognize its importance and are often compelled by the stories of their patients to aim to provide the best treatment’ [P38] and ‘parent advocates and support organisation have become more vocal to ensure that politicians are informed of the concern and asks of bereaved parents and published research evidence on pregnancy has been used to back up these asks and concerns’ [P41]. The benefits of ‘trusted sources disseminating the clinical significance of the research to healthcare practitioners to save them time in assessing the quality of the research’ [P38] was also highlighted.

## Discussion

We aimed to better understand the factors that influence how pregnancy loss research can, or indeed should, influence policy and clinical practice by seeking the views of knowledge users via a qualitative online survey. Participants highlighted specific challenges to translating knowledge regarding pregnancy loss into policy and practice, including stigma, sensitivities and silence, lack of awareness and knowledge, and perceived lack of relevance or priority. These are key issues to attend to and address as part of pregnancy loss knowledge translation activities, in particular knowledge translation for policy change. Lack of knowledge and awareness have been highlighted in previous research [23–26], as has stigma around various forms of pregnancy loss [32–37]. Of interest in our analysis was the perceived stigma within the field itself, with some forms of pregnancy loss being more ‘appropriate’ or easier to communicate around. This finding is also important due to ongoing differences and shifts within definitions of fetal viability which blur the lines between certain types of losses, e.g. second trimester miscarriage and stillbirth [65], and abortion, pregnancy loss, subjective fetal personhood [66] and curtailments to abortion care which also impact on miscarriage care, as well as stigma associated with terminology around ‘miscarriage’ and ‘abortion’ [51, 67]. Appreciating how connected pregnancy loss experiences are will help to normalize and de-stigmatize all pregnancy endings that do not result in a live birth [51, 67]. Furthermore, effective communication is an important part of shaping people’s experiences and views of pregnancy loss, and thus establishing the most appropriate language and framing to use in knowledge translation activities is vital [68–70]. As highlighted elsewhere, cultural, societal and religious barriers to knowledge translation prevail in the field [31].

In the second theme, strategies to enhance the translation of research evidence into policy and practice were highlighted, including making information accessible, engaging and relevant, as well as using a variety of tailored approaches to meet the needs of a broad range of knowledge users. These included materials, workshops/webinars, media, knowledge brokers and champions or opinion leaders. Tailoring interventions to identified barriers may enhance knowledge translation efforts. For example, a Cochrane review found that tailored interventions may improve professional practice compared with no intervention or guideline dissemination alone [71]. Specifically tailoring to knowledge users and the contexts within which they operate is important to enhance effectiveness [72]. Evidence is building around the potential impact of knowledge brokers [73–76] and champions [77, 78] in knowledge translation efforts. Participants highlighted the potential of narratives and the sharing of lived experiences of pregnancy loss to enhance awareness and affect change in policy and practice. This is a potentially powerful knowledge translation strategy [79, 80], with opportunities to develop our understanding of the effectiveness and mechanisms of action of this approach [81]; similar applies to arts-based strategies [82, 83]. The knowledge translation strategies prioritized by knowledge users should be utilized, and evaluated, given that initiatives to improve research-policy engagement in general, while numerous, are poorly evidence informed [84], described and evaluated [85]. The latter can contribute to waste and harms—such as misused time and resources, reduced goodwill towards researchers and increased competition between initiatives [85]. Contextually sensitive strategies are needed, with guidance available on the selection and tailoring of strategies [86, 87]. Particular attention should also be paid to strategies to support the use of evidence in policymaking and the implementation of evidence-based policies, drawing on models such as the Exploration, Preparation, Implementation and Sustainment (EPIS) framework [88] to enhance health policy’s role in dissemination and implementation. The selection of knowledge translation strategies should consider both implementation and sustainability of evidence-based interventions to ensure use and benefit from these, and continue to do so [89].

Allied to international, and indeed national, calls to address issues relating to miscarriage and stillbirth [11, 12], there is also an opportunity to harness the political attention that pregnancy loss is slowly garnering. For example, many countries, including Ireland, are examining supports (including statutory leave) for people who experience pregnancy loss in the workplace particularly following the introduction of paid bereavement leave for couples who experience miscarriage in New Zealand [90], and the United States is grappling with the fall out from the overturning of the constitutional right to abortion care [91], amongst other issues. These are ‘windows of opportunity’ which we can capitalize on in our knowledge translation efforts. Our analysis provides insights to maximize these opportunities, which requires an understanding of the context within which activities or decision making takes place, including societal structures and policy-/decision-making environment [92]. This is an important component of knowledge translation models and frameworks [44–47], which should be used to inform the conduct and evaluation of activities. Knowledge translation is a highly relational process [93]. While published evidence to support best practices is limited [94, 95], meaningfully involving knowledge users, particularly people with lived experience, in both the production and translation of knowledge—through integrated knowledge translation and related approaches (e.g. co-production)—is essential [94, 96, 97].

### Strengths and limitations

Strengths of our work include the inclusion of a diverse range of knowledge users; often research tends to focus on policy/decision makers. This current work involved knowledge users that we engage with in our work at the Pregnancy Loss Research Group, across all types of pregnancy loss; future endeavours could include a broader range of knowledge users, including those internationally. Within the survey we asked knowledge users about barriers and facilitators to the use of research evidence and preferred knowledge translation strategies for pregnancy loss research in general, investigating differences by knowledge user type and intention (practice or policy levels) in our analysis. Further work may be needed to develop knowledge translation plans, with refined knowledge translation strategies, for specific interventions.

While qualitative surveys can be limited by ‘thin responses’, we developed our survey following guidance to mitigate this [98, 99] and had sufficient richness to address our aims. Furthermore, we included data from 46 participants which is a good sample size for a qualitative survey [56]. We provide detailed description of our methodology and illustrative quotes throughout this article to support rigour and to aid reader interpretation of our analysis and its transferability [100].

## Conclusions

Our analysis provides rich insights into the influences on knowledge translation in the field of pregnancy loss. The stigma, sensitivities and silence, lack of awareness and understanding, and lack of relevance or priority afforded to pregnancy loss must be addressed as part of efforts to affect change in practice and policy. Relevant, accessible and engaging information, resources or materials, and tailored approaches that meet the needs of different types of knowledge users are needed, and must be co-created with them. We identified strategies that can be used to inform knowledge translation planning for pregnancy loss research in Ireland, and which have international applicability.

### Supplementary Information

**Additional file 1. **Survey tool

**Additional file 2. **Standards for reporting qualitative research checklist

**Additional file 3: Table S1.** Participant roles: individual roles

## Data Availability

Knowledge users who participated in this involvement activity did not give written consent for their data (beyond anonymized quotes) to be shared publicly. Supporting data is not therefore available, due to the sensitive nature of the activity; however, we provide illustrative quotes throughout this article to support rigour.
