# Supplementary material for: Bridging the gap between pregnancy loss research and policy and practice: insights from a qualitative survey with knowledge users
Source: Health Res Policy Syst. 2024 Jan 25;22:15. doi: 10.1186/s12961-024-01103-z (PMC10809434; doi:10.1186/s12961-024-01103-z)
Supplement: Supplementary file 1 — Additional file 1. Survey tool [file 12961_2024_1103_MOESM1_ESM.docx]

**Additional File 1 Survey**


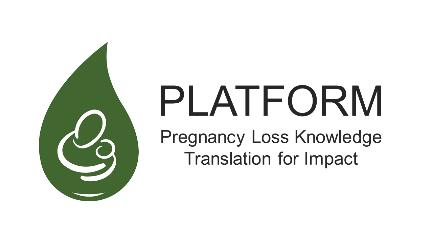


**About this survey - Improving how research is used to influence policy and practice**

We are a multi-disciplinary team of researchers and health/allied health professionals from the Pregnancy Loss Research Group based at Cork University Hospital and University College Cork.

As part of the PLATFORM (Pregnancy Loss knowledge trAnslaTion FOR iMpact) Project, we are bringing researchers and people working or interested in, or who have experienced, pregnancy loss together to help us better communicate and share our work. We want to improve how research evidence is used to influence policy and practice.

Through the PLATFORM Project, we hope to enhance Irish public and policymaker awareness of pregnancy loss, the provision of more effective services/supports within the health care system, community sector, and beyond, and influence policymaking and healthcare funding.

PLATFORM is funded by the Irish Research Council, led by Professor Keelin O’Donoghue in partnership with the Irish Hospice Foundation.

***We need your help***

As someone who engages with us/our work, we would like your help to better understand the factors that influence how pregnancy loss research can, or indeed should, influence policy and practice. We are specifically interested in Ireland, but if there are lessons we could learn from elsewhere, please include these in your responses.

This project focuses on all types of pregnancy and infant loss (including first trimester miscarriage, second trimester miscarriage, ectopic pregnancy, molar pregnancy, stillbirth, termination of pregnancy, neonatal death and pregnancy after loss), and research that seeks to prevent pregnancy loss and improve health care (quality) and health and social outcomes for women, babies and their families.

***What we are asking you to do***

We are asking you to complete this qualitative survey which involves answering ten questions, most of which are open-ended. How long it will take to complete will depend on how much information you would like to share with us. The survey is anonymous; however, you can leave your name/email address if you wish to be contacted about your responses (if you do so, you will not be identified in any write-up of the responses).

We will generate themes from the responses received and use them to develop activities and outputs to improve the use of research evidence in policy and practice. Your responses, or selected quotes from them, may form part of a report to our funders (the Irish Research Council) and other publications/outputs. You will not be identified in any such documents; the quotes will be anonymous. We will keep your original survey responses for the duration of the grant (nine months), after that they will be deleted.

***How to complete the survey***

Please tell us as much as you can for each question – write what immediately comes into your head and/or take time to reflect on what you want to say. Think about your own views/needs, and also reflect on how your colleagues/peers/others might respond to questions.

If you need time to think about the questions/your responses, you can leave the survey and come back to it again; you don’t have to complete it in one sitting. If you leave the survey, your responses will be automatically saved, and the survey link will allow you to return to where you left off (on the same device). You can return to edit your previously completed responses at any stage before submitting the completed version. We will collect partial responses by the closing date (i.e. if you have started your response but have not fully completed the survey).

**If you have any questions** about the survey at any stage, please contact Marita Hennessy, Postdoctoral Researcher within the Pregnancy Loss Research Group ([maritahennessy@ucc.ie](mailto:maritahennessy@ucc.ie))

1. **Which of the following best describes you?** *Please select as many as apply*

- Academic – medical/nursing/midwifery
- Academic – social sciences
- Decision-maker (A person with power to influence or determine policies and practices at an local, regional, national or international level)
- Health professional – community / primary care
- Health professional – hospital-based
- Journalist / media representative
- Medical student
- Parent advocate / bereaved parent
- Policymaker
- Political representative
- Support group representative
- Researcher (including PhD students)
- Other, please specify: _____________________

1. **Who, if any, do you think could benefit from knowing about pregnancy loss research?** *Please select as many as apply*

- Decision-makers (People with power to influence or determine policies and practices at an local, regional, national or international level)
- Media
- Members of the public
- Policy-makers/Government
- Practitioners/service providers, please specify any particular types: ______________
- Private sector/industry
- Research funders
- Researchers
- Volunteer health sector/NGOs
- Women/men with lived/living experience of pregnancy loss
- Other, please specify: _____________________

1. **Please tell us as much as you can about why you selected the particular groups above.** *Think about what you think they should know, and why they should know it.*
2. **Thinking specifically about pregnancy loss: what do you think are barriers, if any, to the use of research evidence by knowledge users?** *Please tell us as much as you can about these barriers, including what they are, why you think they are barriers, and if they are specific to certain types of knowledge users or not. If you can, tell us if certain barriers are greater than others, and why you think that might be.*

Note: Knowledge users include researchers, practitioners/service providers, members of the public, people with lived experience/patients, media, decision-makers, policy-makers/Government, private sector/industry, research funders, volunteer health sector/NGOs.

1. **Again, thinking specifically about pregnancy loss: what enables/facilitates the use of research evidence by knowledge users?** *Please tell us as much as you can about these facilitators, including what they are, why you think they are facilitators, and if you think they are specific to certain types of knowledge users or not. If you can, tell us if certain facilitators are of greater importance than others, and why you think that might be.*

Note: Knowledge users include researchers, practitioners/service providers, members of the public, people with lived experience/patients, media, decision-makers, policy-makers/Government, private sector/industry, research funders, volunteer health sector/NGOs.

1. **Do you think getting pregnancy loss research into policy/practice is any easier or harder than other health topics? Are any aspects of pregnancy loss research easier or harder to get into policy/practice than others?** *Please tell us as much as you can about this, and especially why you think this.*
2. **Would you like pregnancy loss research to be shared with you through…?** *Please select all strategies that apply*

- Knowledge brokers (individuals who link decision-makers and researchers, and facilitate the use of research-based evidence in decision-making)
- Champions / opinion leaders
- Consultants (medical)
- Consultants (non-medical, e.g. business, innovation, marketing)
- Leadership (through leaders who can foster/facilitate change and innovation)
- Collaborations / partnerships
- Materials (guides/toolkits/pamphlets)
- Policy briefs
- Stakeholder position papers
- Grey literature, e.g. reports, working papers
- Peer-reviewed publications, i.e. journal articles
- Workshops, including webinars
- Conferences
- Professional development
- In-service training
- Networks, communities
- Media
- Science policy fellowships, placements
- Meeting dialogue
- Social media. Please specify any particular channels: ____________________
- Arts based strategies, e.g. visual arts, performing arts, creative writing, multimedia-including video and photography
- Other, please specify: ___________________________

1. **Thinking about the last question, do you think certain strategies are more suited to different types of knowledge users?** *Please tell us more about this, including what strategies might suit particular knowledge users (or not) and why you think this. Reflect on what strategies you think work best for you, as well as others here.*
2. **As part of the PLATFORM Project we will be developing a website to provide a platform for sharing information about our Group (The Pregnancy Loss Research Group) and research activities. What information would you like to see on this website?** *Please tell us as much as you can, including what information you think it should contain, how information should be presented and how the website should be structured/laid out to maximise accessibility, or anything else you think is important for us to consider.*
3. **If there is anything else you would like to tell us, please note it here:**

__________________________________________________________________________________

**If you would be happy for us to contact you about your responses, please note your name/email address here. You don't have to provide your details unless you want to.** __________________________________

**Thank you for your time spent taking this survey.**

**Your response has been recorded.**

[Re-direct to PLRG website]
