# Supplementary material for: Bridging the gap between pregnancy loss research and policy and practice: insights from a qualitative survey with knowledge users
Source: Health Res Policy Syst. 2024 Jan 25;22:15. doi: 10.1186/s12961-024-01103-z (PMC10809434; doi:10.1186/s12961-024-01103-z)
Supplement: Supplementary file 3 — Additional file 3: Table S1. Participant roles: individual roles [file 12961_2024_1103_MOESM3_ESM.docx]

**Bridging the gap between pregnancy loss research and policy and practice: Insights from a qualitative survey with knowledge users**

**Table S1 Participant roles: individual roles**

| Role | n | % (N=46) |
| --- | --- | --- |
| Health professional – hospital-based | 16 | 35 |
| Researcher (including PhD students) | 9 | 20 |
| Parent advocate / bereaved parent | 8 | 17 |
| Academic – medical/nursing/midwifery | 7 | 15 |
| Health professional – community / primary care | 6 | 13 |
| Support group representative | 4 | 9 |
| Other | 3 | 7 |
| Journalist / media representative | 3 | 7 |
| Academic – social sciences | 2 | 4 |
| Decision-maker (A person with power to influence or determine policies and practices at local, regional, national or international level) | 2 | 4 |
| Medical student | 1 | 2 |
